# Comprehensive profiling of cell‐free RNA in seminal plasma reveals functional links to spermatogenic impairment

**DOI:** 10.1002/ctm2.70788

**Published:** 2026-09-01

**Authors:** Qing Zhou, Yuting Jiang, Fagui Yue, Chaoxing Wang, Tingyu Yang, Yulong Qin, Chengbin Hu, Zhongzhen Liu, Yuwei Liu, Jinghua Sun, Fang Chen, Ya Gao, Xin Jin, Ruizhi Liu, Wen‐Jing Wang, Hongguo Zhang

**Affiliations:** ^1^ State Key Laboratory of Genome and Multi‐omics Technologies BGI Research Shenzhen China; ^2^ Shenzhen Key Laboratory of Transomics Biotechnologies BGI Research Shenzhen China; ^3^ Reproductive Medicine Center Prenatal Diagnosis Center First Hospital of Jilin University Changchun China; ^4^ College of Life Sciences University of Chinese Academy of Sciences Beijing China; ^5^ Shenzhen Engineering Laboratory for Birth Defects Screening Shenzhen China

To the Editor,

We provide a systematic multi‐class cell‐free RNA (cfRNA) atlas of seminal plasma, establishing it as a powerful, non‐invasive strategy that both elucidates molecular mechanisms underlying spermatogenic impairment and offers candidate biomarkers to optimize the outcome of assisted reproductive technology (ART) before treatment for infertile men.

Seminal plasma is a promising, non‐invasive source of biomarkers for diagnosing and treating male infertility. However, the absence of systematic characterization of seminal cfRNA hinders our understanding of the full molecular diversity in seminal plasma and limits the development of clinical strategies for male infertility.

We established a comprehensive multi‐class cfRNA atlas of human seminal plasma from 419 men, including 101 healthy individuals, 128 with reduced sperm motility (defined as AZS), 93 with reduced sperm number and motility (defined as OAS), and 97 with azoospermia (AZO) (Figure 1A; Figure S1; Tables S1 and S2; Materials and Methods). After quality control (Figure S2), we robustly detected diverse small and long RNA in human seminal plasma (Table S3). Long RNA (mRNA and lncRNA) constituted the largest proportion, followed by transfer RNA (tRNA) and piwi‐RNA (piRNA) (Figure 1B). For microRNA (miRNA), we also captured biologically relevant species (Figure S3). The fragments of 17–33 nucleotides with a strong bias for a 5′‐terminal uridine (1U bias) provided evidence for the existence of mature piRNA in seminal plasma (Figure 1C), and the most abundant tRNA and their fragmentation patterns were consistent with those in mouse and human sperm (Figure 1D,E; Figure S4).^1^, ^2^ Thus, seminal cfRNA offers a unique window into the dynamic RNA payload switch, whereby epididymosomes deliver tRNA fragments that largely replace piRNA during epididymal transit.^3^ By deconvoluting the testicular cellular origins of seminal cfRNA, macrophages showed the highest contribution score (Figure 1F; Table S4), aligning with the known presence of immune modulators in seminal plasma.^4^ We also detected significant signals from germ cells across multiple stages, indicating the release of RNA from the entire spermatogenic lineage. Our findings suggest seminal plasma as a rich source of molecular information reflecting testicular microenvironment dynamics.

**FIGURE 1:**
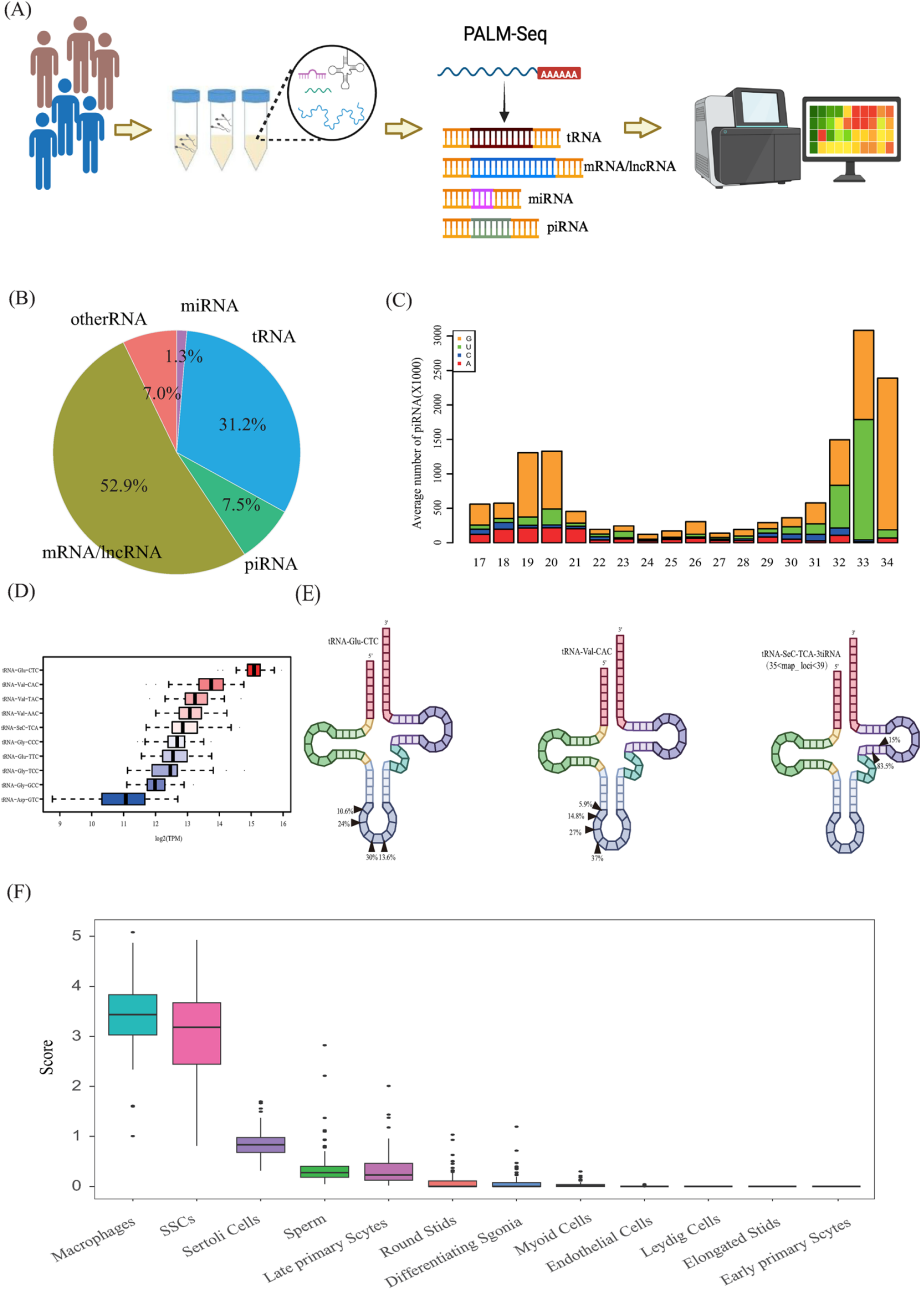
Comprehensive profiling of cell‐free RNA in human seminal plasma. (A) Overview of participant recruitment, sample processing, and library construction. Cell‐free RNA (cfRNA) sequencing was performed using polyadenylation ligation‐mediated sequencing (PALM‐Seq). (B) Composition of the seminal cfRNA library from healthy individuals. Pie chart depicts the relative abundance of different RNA classes. (C) Length distribution and 5′‐terminal nucleotide bias of seminal piRNA. Height of bar represents fragment count for each length and colour indicates the proportion of each nucleotide (A, U, C, G) at the 5′‐most position. (D) The top ten tRNAs with most abundance in seminal plasma. Box plots display the level (RPM) for each tRNA. (E) Schematic representation of the most abundant tRNA‐derived fragments (tRFs). Fragment length and relative abundance are shown for the dominant tRNA species; arrows mark predominant 3′ cleavage sites. (F) Cellular deconvolution of testicular contributions to seminal cfRNA. Box plots show the estimated proportion (contribution score) of each testicular cell type derived using CIBERSORTx.

Next, we observed distinct cfRNA composition shifts across infertility subtypes, most notably in AZO patients across all RNA classes (Figure 2A). Differential abundance analysis identified hundreds of altered cfRNA (Figure 2B; Table S5, adjusted *p*‐value < .05 and |log_2_(fold change)| ≥ 1), with declined mRNA involving core spermatogenic functions, including piRNA metabolic pathways (Figure 2C,D). Strikingly, pachytene piRNA was preferentially decreased in patients with AZS and a substantial fraction became undetectable in patients with AZO (Figure 2E). In addition, altered piRNA lacked canonical 1U bias (Figure 2F–D; Figure S5), suggesting piRNA abnormalities consistent with impaired piRNA biogenesis or regulation. Weighted gene co‐expression network analysis revealed seminal cfRNA strongly correlated with crucial parameters of sperm quality (Figure 2H; Figure S6 and S7) and genes within the correlated module were predominantly involved in male gamete generation (Figure S8). Cellular deconvolution showed that signals from late primary spermatocytes and round spermatids were markedly reduced in infertile men (Figure 2I; Figure S9), recapitulating known histopathological changes of spermatogenic arrest. Collectively, seminal cfRNA signatures not only distinguish between different infertility phenotypes but also recapitulate the cellular composition changes within the testis, inferring spermatogenic impairment.

**FIGURE 2:**
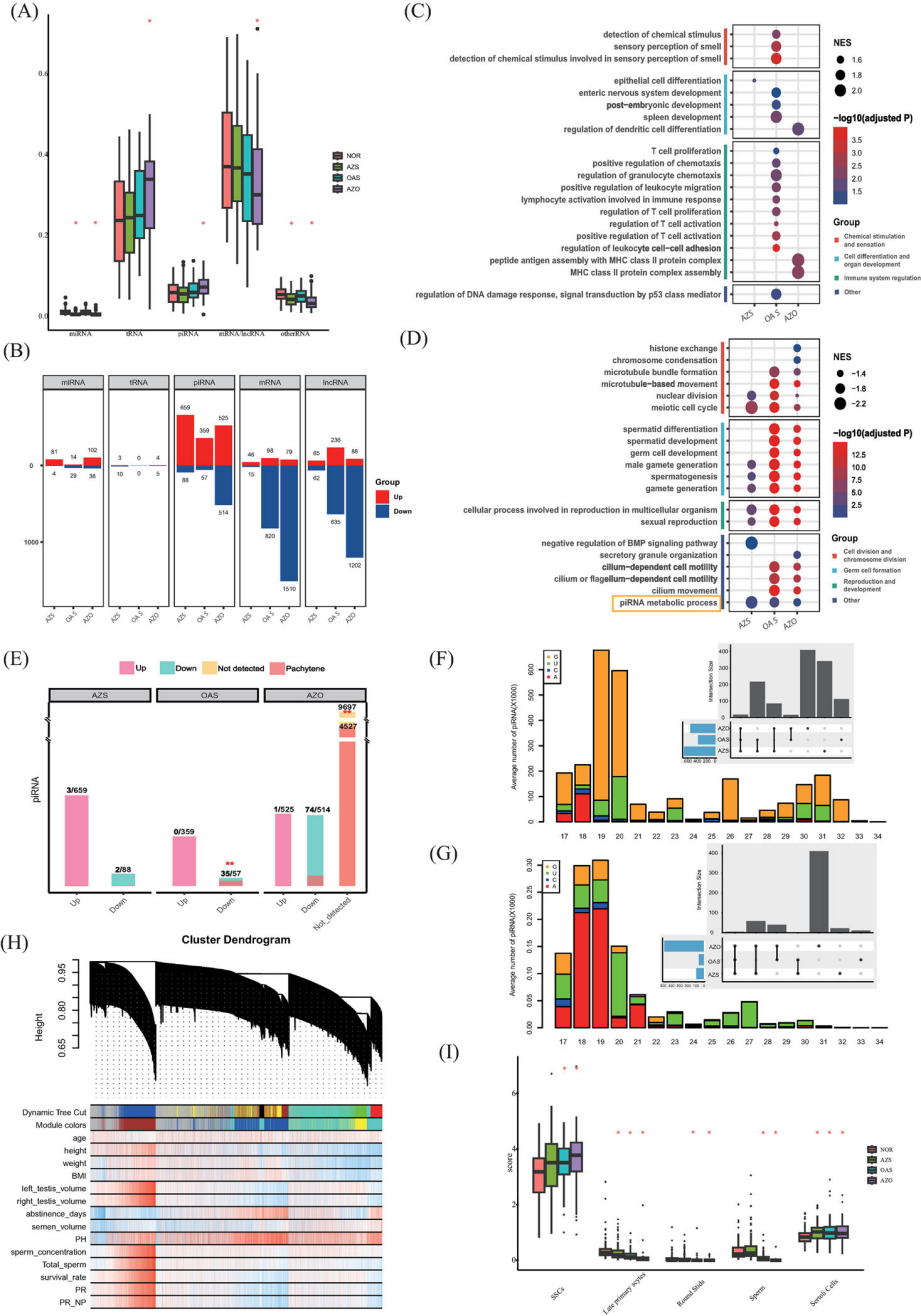
Dysregulation of seminal cfRNA landscapes in men with spermatogenic impairment. (A) Compositional shifts of major cfRNA classes across infertile groups. Box plots compare the relative abundance of each RNA class in normozoospermic (NOR) men and patients with lower sperm motility (AZS), lower sperm count (OAS), or azoospermia (AZO). Significant differences (*p* < .01, Wilcox Rank Sum test) are marked with a red asterisk. (B) Number of differentially abundant cfRNA in each infertile group versus NOR controls. Increased and decreased cfRNA are shown in red and blue, respectively. (C, D) Gene Set Enrichment Analysis (GSEA) of increased (C) and decreased (D) mRNA in infertile men. Enriched biological processes are displayed and grouped by similarity. (E) Altered piRNA populations in infertility. Bar chart shows the total number of dysregulated piRNA per group, with the subset belonging to the pachytene class highlighted in orange. **p < .001. (F, G) Characteristics of increased (F) and decreased (G) piRNA. Upset plots (right) illustrate overlaps of altered piRNA across the three infertile groups. Associated bar charts depict the length distribution and 5′‐terminal nucleotide composition of these piRNA in AZS patients. (H) Co‐expression modules of long RNA (mRNA and lncRNA) identified by weighted gene co‐expression network analysis (WGCNA). The dendrogram clusters transcripts into color‐coded modules. Heatmap rows below indicate the correlation (red, positive; blue, negative) of each module with key clinical traits. BMI: body mass index, PR: progressive motility. (I) Altered testicular cellular contributions to seminal cfRNA in infertility. Box plots show the contribution scores (generated by CIBERSORTx) of four germ‐cell and somatic‐cell types that significantly changed between fertile and infertile men. SSCs, spermatogonial stem cells; Round Stids, round spermatids.

Notably, the vast majority of altered seminal cfRNA in AZO patients were declined, including several established AZO‐associated pathogenic genes like *PNLDC1*, *SPATA22*, and *MEIOB* (Figure 3A).^5^, ^6^, ^7^ These dysregulated RNAs mainly regulate spermatogenesis (Figure 3B). Along with the inverse expression pattern of miRNA, we identified a competing endogenous RNA (ceRNA) network in AZO centred on *hsa‐miR‐125a‐3p*, which targets both mRNA *YBX2* and lncRNA *CDKN2B‐AS1* (Figure 3C–E). *YBX2* encodes a key RNA‐binding protein essential for stabilizing and translating germ cell mRNA, and its loss in mice causes spermatogenic arrest and AZO.^8^ Both *YBX2* and *CDKN2B‐AS1* are highly expressed in human germ cells across developmental stages (Figure 3F), and their expression is significantly reduced in the germ cells of infertile men (Figure 3G).^9^ Thus, our results implicate the *hsa‐miR‐125a‐3p‐YBX2/CDKN2B‐AS1* axis as a potential regulator associated with human spermatogenic failure, showcasing how seminal cfRNA profiles can uncover disease‐relevant molecular interactions.

**FIGURE 3:**
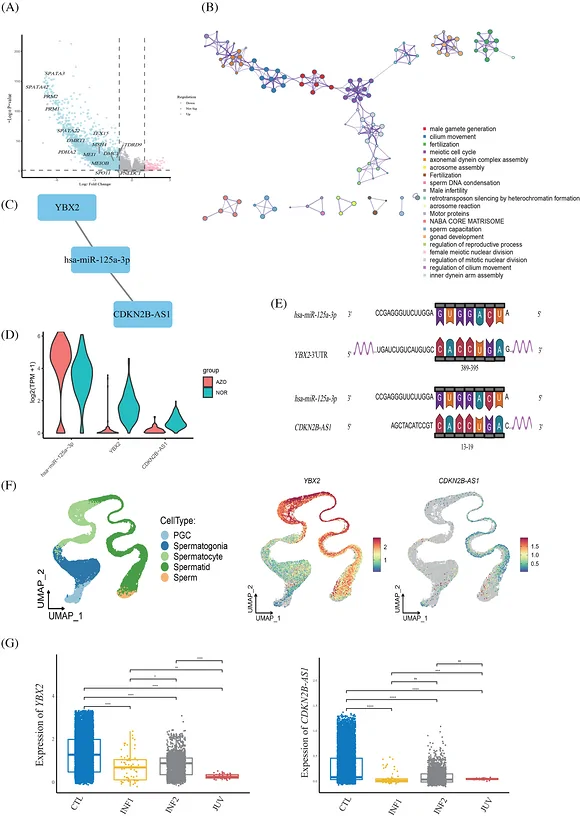
Dysregulated molecular networks in the seminal plasma of men with azoospermia. (A) Differentially abundant seminal cfRNA in azoospermia (AZO) patients versus normozoospermic controls. Volcano plot displays increased (pink) and decreased (blue) transcripts. Labelled points denote known AZO‐associated pathogenic genes. (B) Functional enrichment network of dysregulated mRNA in AZO. Nodes represent enriched biological terms; edges connect terms with overlapping gene sets, with thickness proportional to similarity. Clusters of related functions are spatially grouped. (C) Competing endogenous RNA (ceRNA) network identified from seminal cfRNA of AZO patients. Networks were visualized using Cytoscape and highlight miRNA‐mediated regulatory interactions among lncRNA and mRNA. (D) Violin plots show a higher level of *hsa‐miR‐125a‐3p* and lower abundance of *YBX2* mRNA and *CDKN2B‐AS1* lncRNA in seminal plasma of AZO patients. (E) Predicted binding sites of hsa‐miR‐125a‐3p within the 3′ untranslated region (3′ UTR) of *YBX2* mRNA and the transcript body near the transcript start site of *CDKN2B‐AS1* lncRNA. (F) Single‐cell expression of candidate ceRNA network components in the human testis. Uniform Manifold Approximation and Projection (UMAP) plot shows germ‐cell populations (left), with point colours indicating expression levels of the indicated mRNA‐lncRNA pairs across cell types. PGC, primordial germ cell. (G) Reduced expression of *YBX2* and *CNKN2B‐AS1* in testicular germ cells of infertile men. Boxplot plots compare *YBX2* expression levels across germ cells from a control donor (CTL), an idiopathic azoospermic individual (INF1), a patient with retrograde oligozoospermia (INF2), and a juvenile sample (JUV). Data are derived from a published single‐cell RNA‐seq study ^9^. *p < .01(Wilcox Rank Sum test), **p < .001,***p < .0001,****p < .00001, ns: not significant.

To assess the predictive utility of seminal cfRNA in clinical practice, we employed bootstrapping to identify the most informative features, followed by random forest regression (Figure 4A). We trained models to predict seminal parameters including total sperm number, progressive motility (PR) and total motility (TM), showing high accuracy with *R* values of  .73,  .79, and  .79, respectively (Figure 4B–D, fivefold cross‐validation). The overall prediction accuracy distinguishing normozoospermic men from patients with abnormal sperm number (<39 × 10^6^ per ejaculate) was 85.9%, and we classified AZS patients from controls with an accuracy of 89.1% (Figure 4E).

**FIGURE 4:**
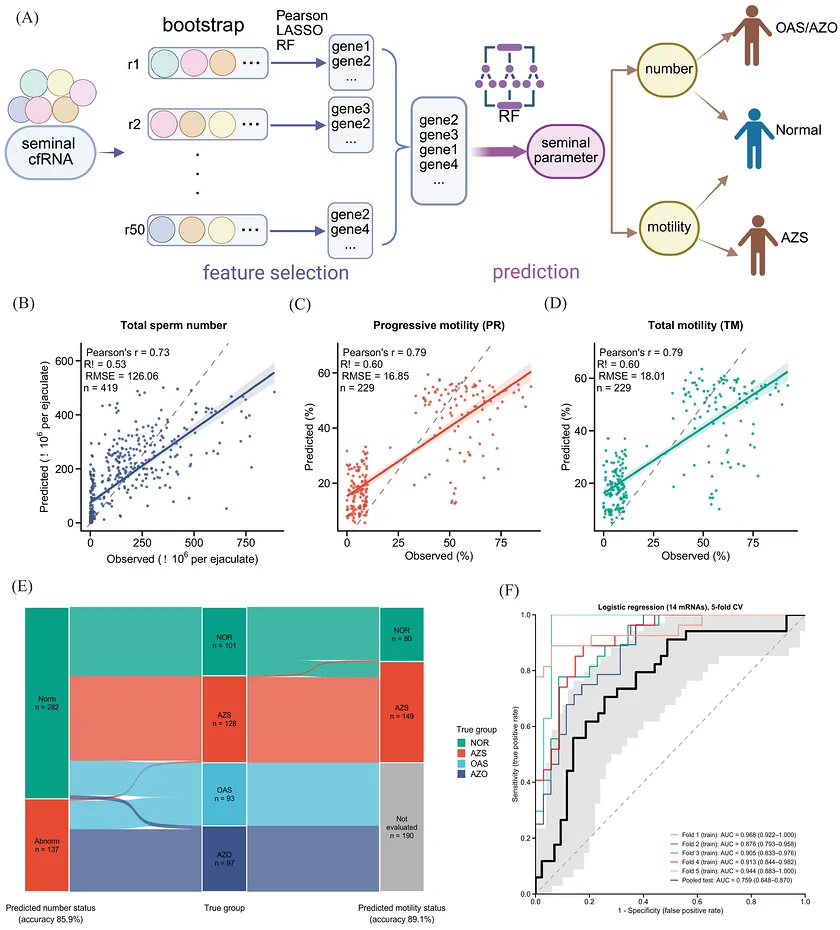
Machine‐learning models for predicting semen parameters and assisted reproductive technology outcome. (A) Workflow for model construction. Key steps include feature selection from seminal cfRNA, training of regression models to predict continuous semen parameters (total sperm number and motility), and classification of individuals based on model outputs. (B–D) Performance of random forest regression models in predicting total sperm number (B), progressive motility (PR) (C), and total motility (TM) (D) via fivefold cross‐validation. Scatter plots show the correlation between predicted and clinically measured values. (E) Sankey diagram showing the classification performance of the predictive model for number and motility status across four groups: NOR, AZS, OAS, and AZO. The middle‐side nodes represent the true group labels, and the left‐ and right‐side nodes represent the model‐predicted assignments based on number (left) and motility (right). The widths of the connecting ribbons are proportional to the number of samples transitioning from each true category to each predicted category. (F)Overall performance of the model in predicting ART outcome. The ROC curve summarizes classification accuracy across fivefold cross‐validation, with the area under the curve (AUC) quantifying predictive power.

Among these patients receiving ART therapy, 34 couples had successful clinical pregnancy, and 43 patients failed through several cycles. An elastic‐net logistic regression model derived from 14 cfRNA signatures achieved a mean area under the receiver operating characteristic curve (AUROC) of  .75 in distinguishing successful from failed ART treatment (Figure 4F, fivefold cross‐validation). High‐contribution features included *TUBB3* and *SAXO1* (Figure S10), both encoding microtubule‐associated proteins critical for early embryonic development. In addition, our predictive system achieved an accuracy of 61.1% (11/18) when identifying ART outcome in a validation group (Figure S10). Therefore, seminal cfRNA not only shows strong associations with semen parameters but also shows preliminary predictive association with ART outcome, pointing to specific molecular pathways that may inform future therapeutic strategies.

In conclusion, we provide a systematic cfRNA atlas of human seminal plasma. We identified piRNA abnormalities as a hallmark of spermatogenic impairment and discovered a putative ceRNA interaction targeting *YBX2* in AZO patients. Moreover, seminal cfRNA showed a strong association with semen parameters and ART outcome. We acknowledge that the heterogeneity in our present cohort may contribute to variability in cfRNA signatures, and the limited number of samples may increase the risk of overfitting. This cross‐sectional study lacks external validation in multi‐centre and trans‐ethnic cohorts. Future stratified analyses in larger cohorts with strictly controlled covariates are needed to dissect the molecular diversity within specific phenotypes. Our study provides a proof‐of‐concept that seminal cfRNA could reflect testicular microenvironment dynamics, advance non‐invasive assessment methods, and suggest directions for future personalized treatment strategies.

## AUTHOR CONTRIBUTIONS

Qing Zhou designed the experiments, performed the major analysis, and wrote the paper. Yuting Jiang and Fagui Yue recruited participants and dealt with clinical data. Tingyu Yang and Chengbin Hu contributed to data analysis and model construction. Chaoxing Wang assisted with model construction during revision. Zhongzhen Liu and Yuwei Liu conducted the experiments. Wen‐Jing Wang and Hongguo Zhang conceived the study and revised the manuscript. Yulong Qin and Jinghua Sun contributed to the analysis. Fang Chen, Ya Gao, Xin Jin, and Ruizhi Liu helped design the experiments and revise the manuscript. All authors have revised the final version of the manuscript and agreed to the submission.

## ETHICS STATEMENT

This study was approved and guided by the Medical Ethics Committee of the First Hospital of Jilin University (20K102‐001). All procedures involving human participants were followed the ethical standards of the institutional committee and in accordance with the 1964 Helsinki Declaration and its later amendments or comparable ethical standards. All participants were recruited from the Reproductive Medicine Center of the First Hospital of Jilin University following written informed consent.

## CONSENT FOR PUBLICATION

The authors have nothing to report.

## CONFLICT OF INTEREST STATEMENT

The authors declare no conflict of interest.

## Supporting information




**Supporting File**: ctm270788‐sup‐0001‐Figure S1‐S10.pdf.

**Supporting File**: ctm270788‐sup‐0002‐Table S1.docx

**Supporting File**: ctm270788‐sup‐0003‐Table S2.xlsx.

**Supporting File**: ctm270788‐sup‐0004‐Table S3.xlsx.

**Supporting File**: ctm270788‐sup‐0005‐Table S4.xlsx.

**Supporting File**: ctm270788‐sup‐0006‐Table S5.xlsx.

**Supporting File**: ctm270788‐sup‐0007‐Materials and Methods.docx

## Data Availability

The raw cfRNA data have been deposited into CNGBdb (https://db.cngb.org/) with accession number CNP0005729. Data will be made available on request. The code for analysis is available at https://github.com/zqyezitang/seminal‐cfRNAs.
