# Supplementary material for: Comprehensive profiling of cell‐free RNA in seminal plasma reveals functional links to spermatogenic impairment
Source: Clin Transl Med. 2026 Sep 1;16(9):e70788. doi: 10.1002/ctm2.70788 (PMC13535125; doi:10.1002/ctm2.70788)
Supplement: Supplementary file 1 [file CTM2-16-e70788-s004.pdf]

Supplementary Figure legends

Figure S1. Pipeline of sample recruitment and classification.

A flowchart shows the principle to collect and classify samples. Finally, individuals with lower sperm motility and normal sperm count were classified as AZS group. Men with no sperm were in AZO group while men with both lower sperm count and motility were defined as OAS. The parameter of sperm morphology was not considered here. The reference threshold for sperm count and motility was based on the 5th edition of the World Health Organization's criteria. PR: Progressive Motility, NP: Non-Progressive Motility.

Figure S2. Quality control of seminal plasma cfRNA sequencing data.

(A) Principal component analysis (PCA) of all sequenced samples based on the expression profiles of miRNA, tRNA, piRNA, mRNA, and lncRNA. Samples with an exceptionally low number of clean reads cluster near the negative control (CTL, water) and were flagged as outliers.

(B) Density distribution of total mapped reads per sample following data preprocessing.

(C) PCA of samples retained after quality control, performed separately for each RNA class (miRNA, tRNA, piRNA, mRNA, and lncRNA).

Figure S3. Abundance and seed- region composition of seminal plasma miRNAs.

(A) Expression levels of selected miRNAs in seminal plasma. Violin plots depict the distribution of normalized read counts (RPM) for *hsa-mir-1246*, *hsa-mir-6075*, and representative spermiogenesis-associated miRNAs.

(B) Nucleotide frequency within the miRNA seed region. The frequency of each nucleotide (A, U, C, G) at positions 2–8 from the 5′ end is shown for detected seminal miRNAs (left) and for random seed sequences generated from all annotated miRNAs (right).

Figure S4. Abundance landscape of merged tRNA isodecoders in seminal plasma.

Heatmap displays the abundance of combined tRNA reads (aggregated by isodecoder) across samples from normozoospermic (NOR) men and patients with lower motility (AZS), lower sperm count (OAS), and no sperm (AZO). Rows represent tRNA isodecoders, columns represent individual samples, and color intensity corresponds to normalized expression level.

Figure S5. Length and 5′-end nucleotide profiles of dysregulated piRNAs in oligozoospermia and azoospermia.

(A, B) Length distribution and 5′-terminal nucleotide composition of piRNAs that are increased (left panels) or decreased (right panels) in men with lower sperm count (OAS) **(A)** and no sperm (AZO) **(B)**. Bar height represents the relative abundance of piRNA fragments at each indicated length; color denotes the proportion of each nucleotide (A, U, C, G) at the 5′-most position.

Figure S6. Weighted gene co-expression network analysis (WGCNA) of seminal miRNAs.

(A) Hierarchical clustering dendrogram of miRNAs grouped into co-expression modules (color-coded branches). The lower heatmap rows indicate the correlation (red, positive; blue, negative) of each module with key clinical traits, including body mass index (BMI) and progressive motility (PR).

(B) Expression heatmap of miRNAs belonging to the module showing the strongest overall correlation with clinical parameters (highlighted in green in panel A). Rows represent individual miRNAs, columns represent samples, and color intensity reflects normalized abundance.

Figure S7. Weighted gene co-expression network analysis (WGCNA) of seminal piRNAs.

(A) Hierarchical clustering dendrogram of piRNAs grouped into co-expression modules (color-coded branches). The lower heatmap rows indicate the correlation (red, positive; blue, negative) of each module with key clinical traits.

(B) Module-trait correlation heatmap. Color intensity and direction (red, positive; blue, negative) represent the correlation coefficient between each module eigengene and clinical features; numbers within cells denote the corresponding p-value.

(C) Abundance heatmap of piRNAs belonging to the “saddlebrown” module, which shows the strongest overall correlation with clinical parameters. Rows represent

individual piRNAs, columns represent samples, and color intensity reflects normalized value of abundance.

(D) Violin plot comparing the level (RPM) of piRNAs in the “saddlebrown” module between normozoospermic (NOR) and infertile (AZS/OAS/AZO) groups.

BMI, body mass index; PR, progressive motility.

Figure S8. Functional annotation and disease association of mRNA in the WGCNA “brown” module.

(A) Significantly enriched biological processes among genes comprising the “brown” co- expression module, as identified by Gene Ontology (GO) analysis.

(B) Human diseases and disorders significantly associated with genes in the “brown” module, based on curated annotations from the DisGeNET database.

Figure S9. Testicular cell- type contributions to seminal cfRNAs across diagnostic groups.

Box plots compare the estimated contribution (origin score) of each testicular cell type to the seminal cfRNA pool among normozoospermic (NOR) men and patients with lower motility (AZS), lower sperm count (OAS), or no sperm (AZO). Origin scores were derived using CIBERSORTx deconvolution analysis.

Figure S10. Feature importance and confusion matrix for the ART- outcome prediction model.

(**A-E**) SHapley Additive exPlanations (SHAP) summary plot shows the direction and

magnitude of each feature's contribution to the predicted probability of ART success (positive SHAP value) or failure (negative SHAP value) in each fold. Color indicates the absolute abundance of the feature. (**F**) Confusion matrix indicates the accuracy of predicting models on an independent validation group.

FigS1

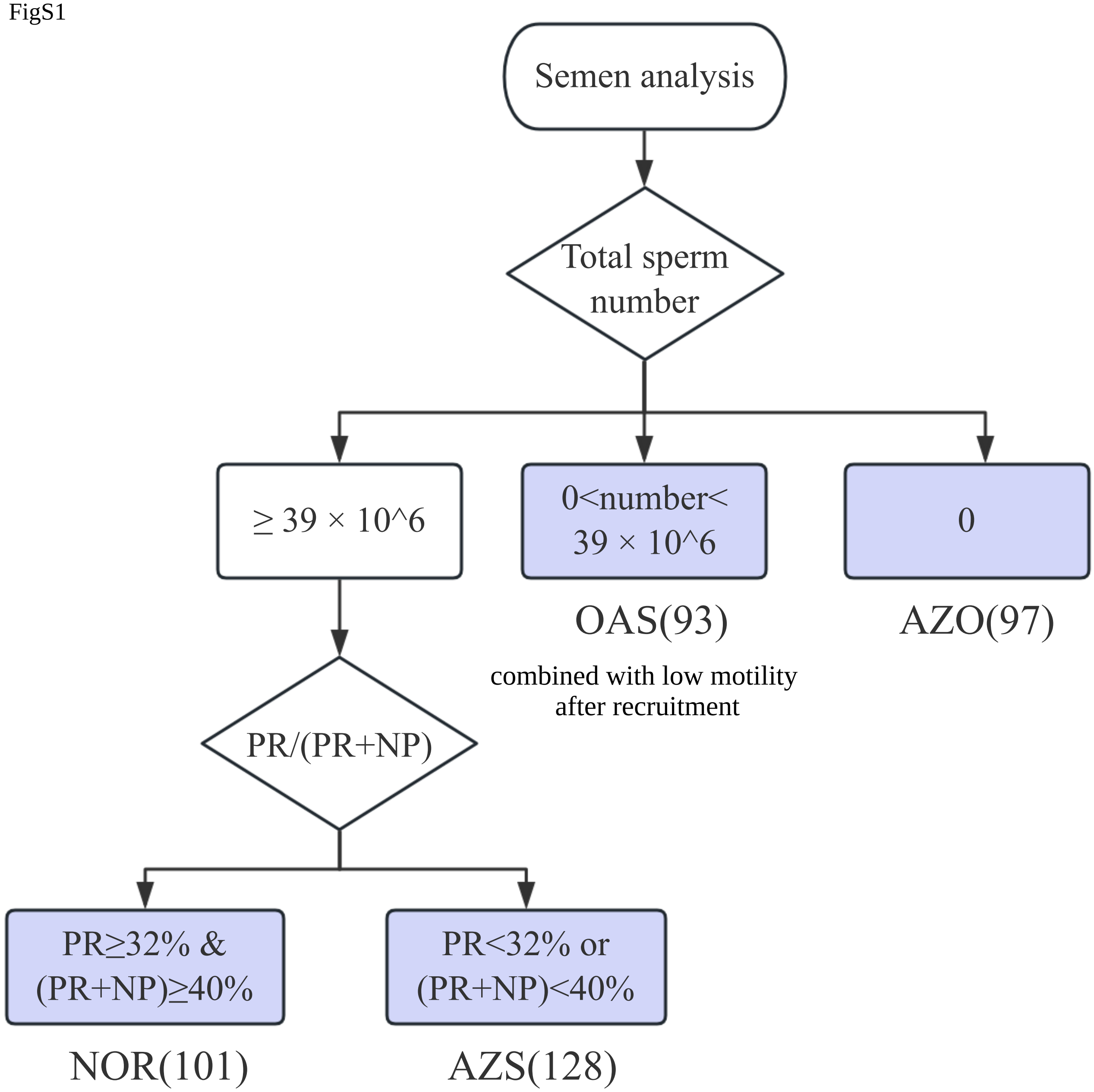
Semen analysis
Total sperm number
≥ 39 × 10^6
0<number< 39 × 10^6
0
OAS(93)
AZO(97)
combined with low motility after recruitment
PR/(PR+NP)
PR≥32% & (PR+NP)≥40%
PR<32% or (PR+NP)<40%
NOR(101)
AZS(128)

FigS2

A

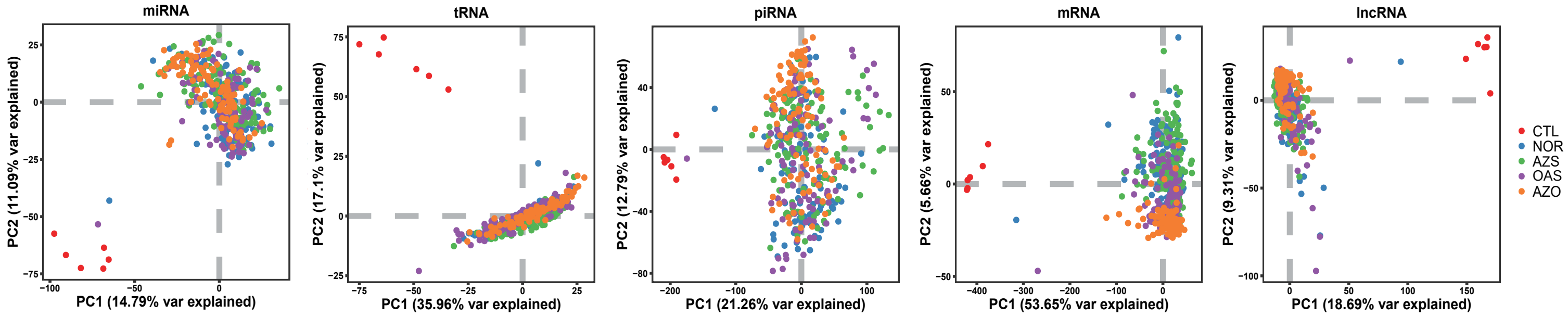
miRNA
PC2 (11.09% var explained)
PC1 (14.79% var explained)
tRNA
PC2 (17.1% var explained)
PC1 (35.96% var explained)
piRNA
PC2 (12.79% var explained)
PC1 (21.26% var explained)
mRNA
PC2 (5.66% var explained)
PC1 (53.65% var explained)
lncRNA
PC2 (9.31% var explained)
PC1 (18.69% var explained)
CTL
NOR
AZS
OAS
AZO

B

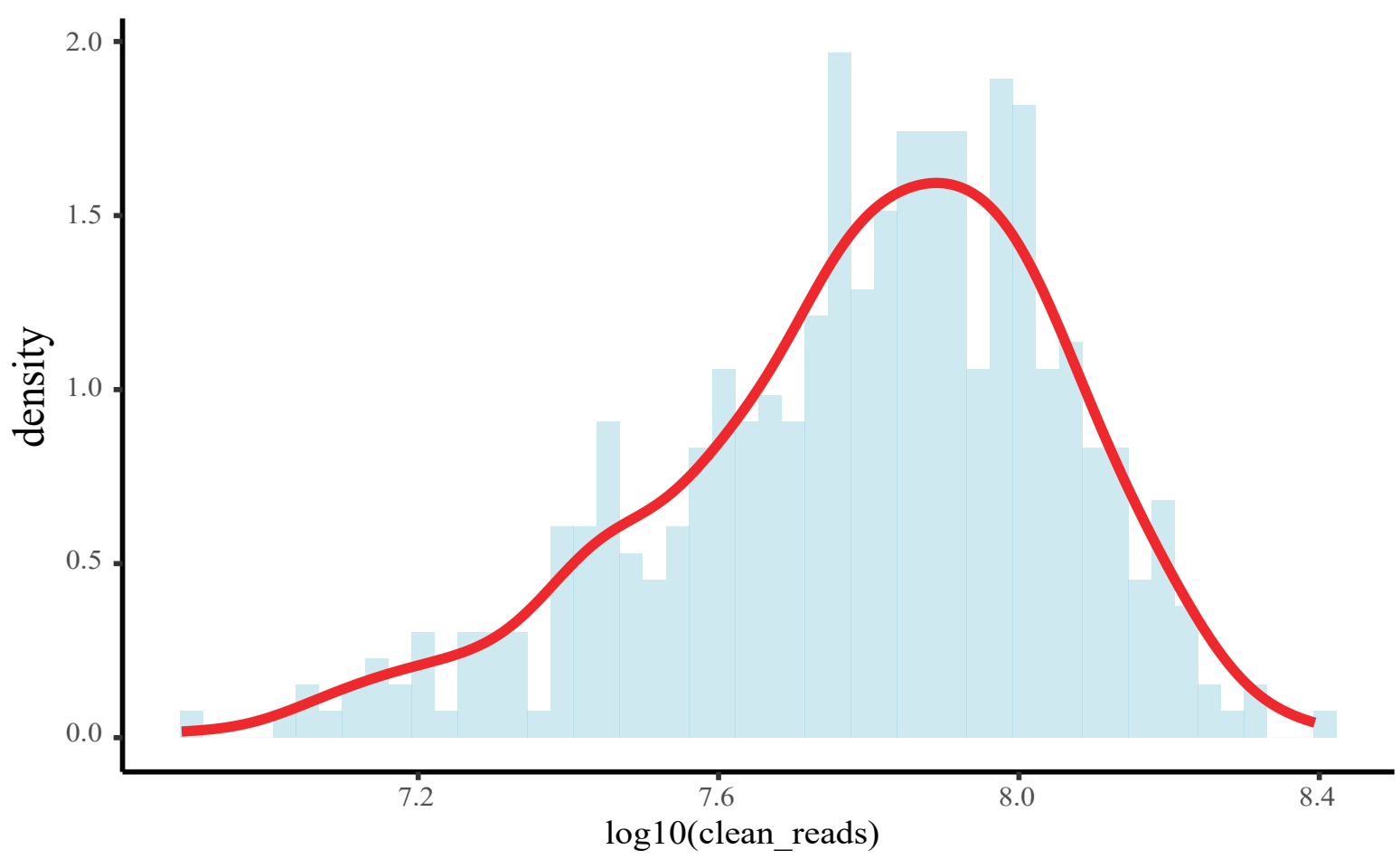
density
log10(clean_reads)

C

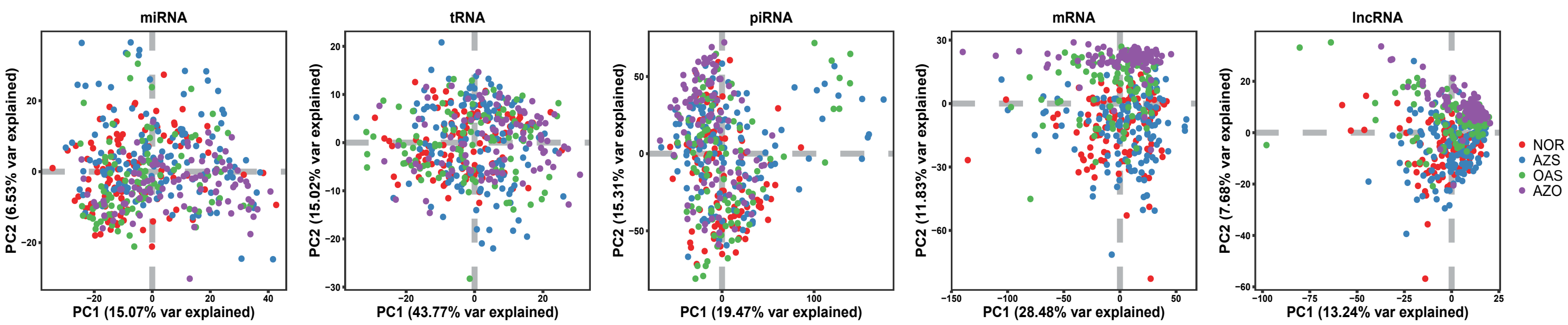
miRNA
PC2 (6.53% var explained)
PC1 (15.07% var explained)
tRNA
PC2 (15.02% var explained)
PC1 (43.77% var explained)
piRNA
PC2 (15.31% var explained)
PC1 (19.47% var explained)
mRNA
PC2 (11.83% var explained)
PC1 (28.48% var explained)
lncRNA
PC2 (7.68% var explained)
PC1 (13.24% var explained)
NOR
AZS
OAS
AZO

FigS3

A

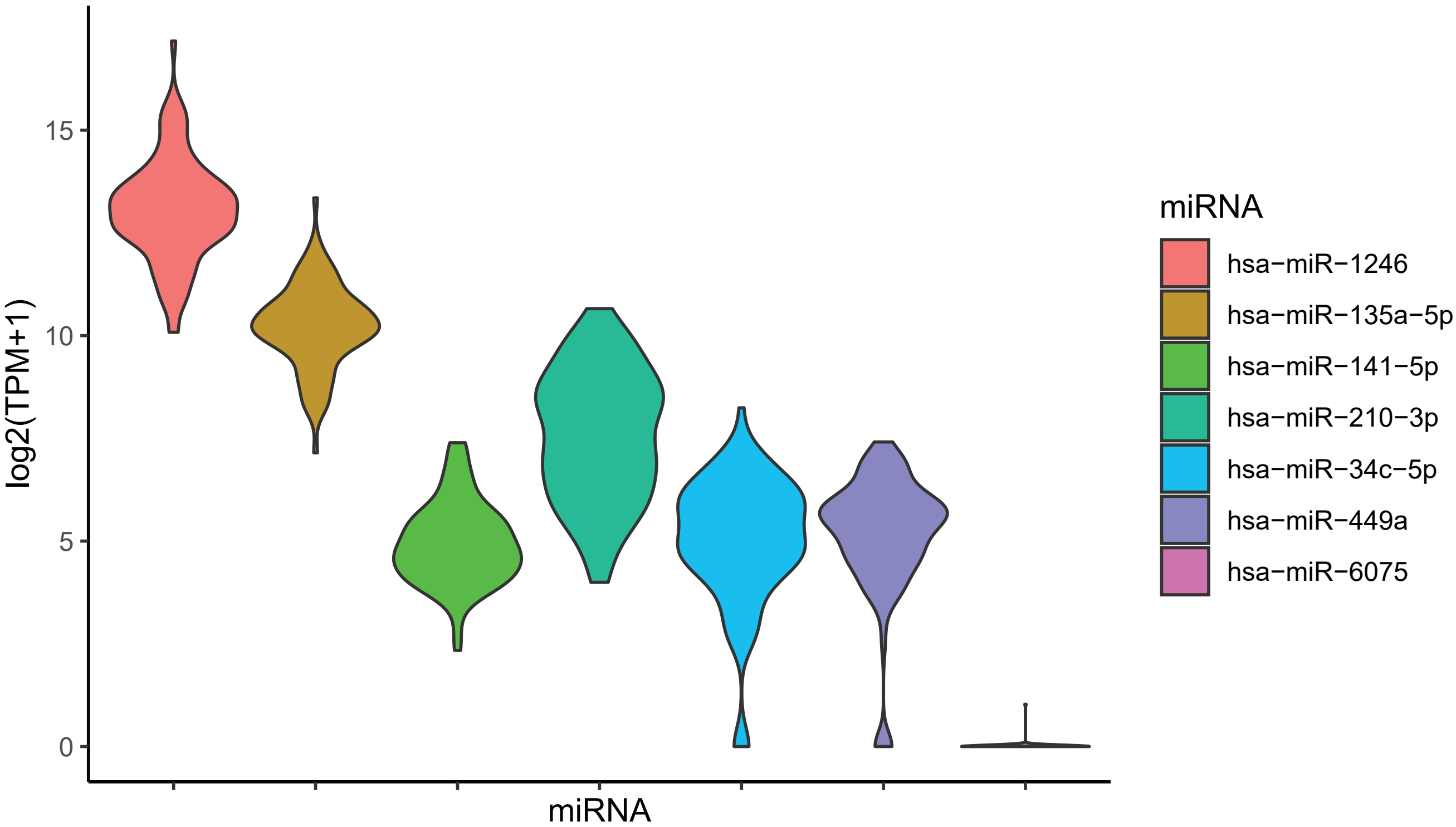

log2(TPM+1)
15
10
5
0
miRNA
miRNA
hsa−miR−1246
hsa−miR−135a−5p
hsa−miR−141−5p
hsa−miR−210−3p
hsa−miR−34c−5p
hsa−miR−449a
hsa−miR−6075

B

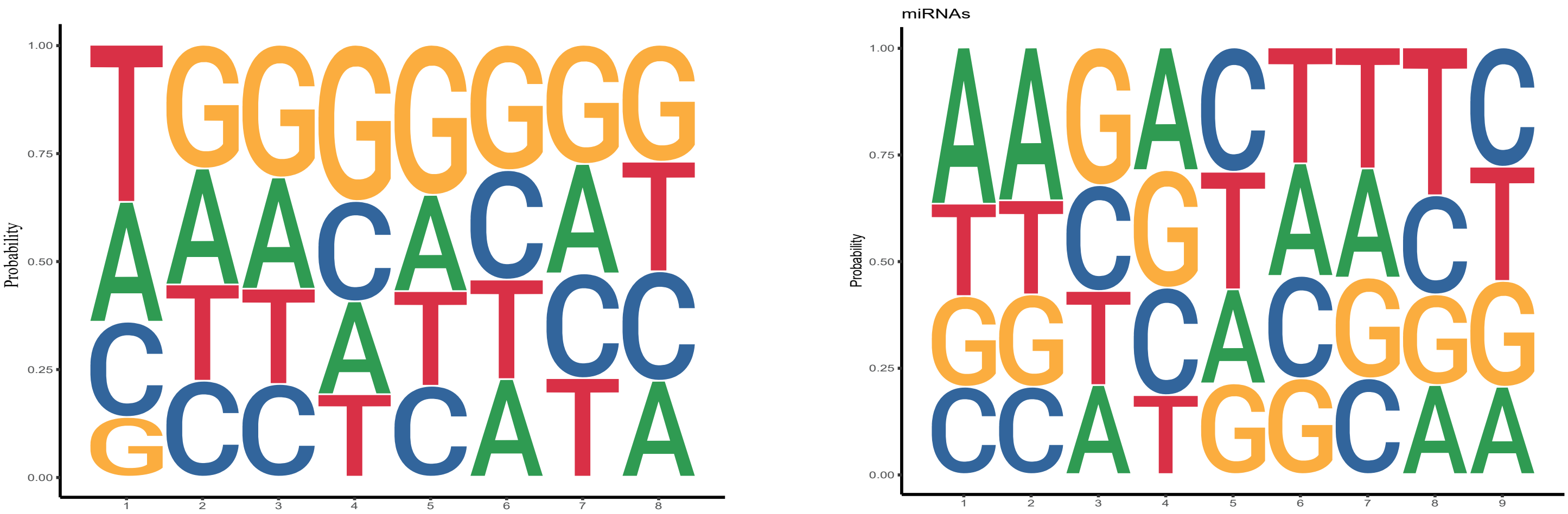

Probability
1.00
0.75
0.50
0.25
0.00
1
2
3
4
5
6
7
8
miRNAs
Probability
1.00
0.75
0.50
0.25
0.00
1
2
3
4
5
6
7
8
9

FigS4

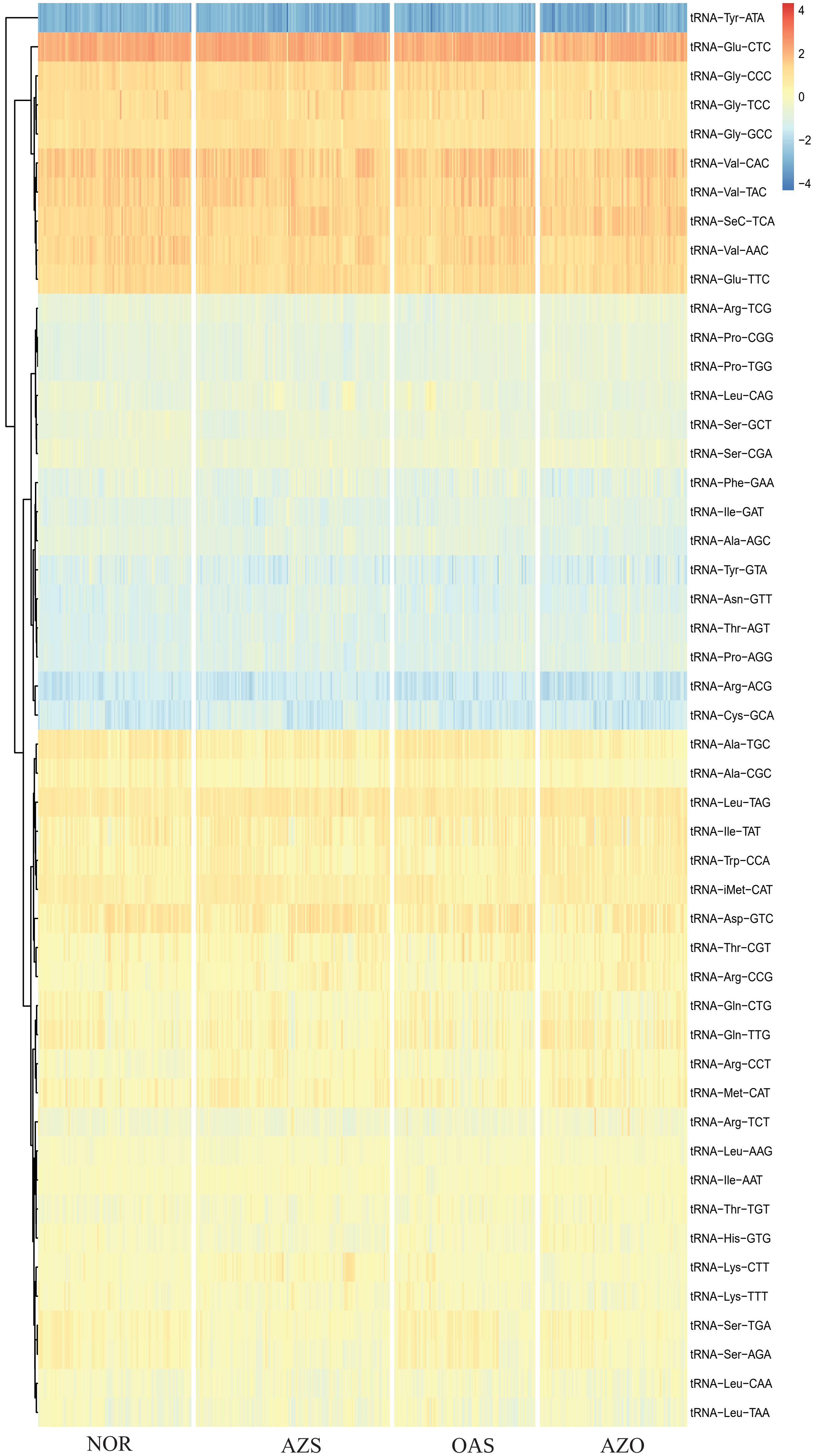
tRNA-Tyr-ATA
tRNA-Glu-CTC
tRNA-Gly-CCC
tRNA-Gly-TCC
tRNA-Gly-GCC
tRNA-Val-CAC
tRNA-Val-TAC
tRNA-SeC-TCA
tRNA-Val-AAC
tRNA-Glu-TTC
tRNA-Arg-TCG
tRNA-Pro-CGG
tRNA-Pro-TGG
tRNA-Leu-CAG
tRNA-Ser-GCT
tRNA-Ser-CGA
tRNA-Phe-GAA
tRNA-Ile-GAT
tRNA-Ala-AGC
tRNA-Tyr-GTA
tRNA-Asn-GTT
tRNA-Thr-AGT
tRNA-Pro-AGG
tRNA-Arg-ACG
tRNA-Cys-GCA
tRNA-Ala-TGC
tRNA-Ala-CGC
tRNA-Leu-TAG
tRNA-Ile-TAT
tRNA-Trp-CCA
tRNA-iMet-CAT
tRNA-Asp-GTC
tRNA-Thr-CGT
tRNA-Arg-CCG
tRNA-Gln-CTG
tRNA-Gln-TTG
tRNA-Arg-CCT
tRNA-Met-CAT
tRNA-Arg-TCT
tRNA-Leu-AAG
tRNA-Ile-AAT
tRNA-Thr-TGT
tRNA-His-GTG
tRNA-Lys-CTT
tRNA-Lys-TTT
tRNA-Ser-TGA
tRNA-Ser-AGA
tRNA-Leu-CAA
tRNA-Leu-TAA
4
2
0
-2
-4
NOR
AZS
OAS
AZO

FigS5

A

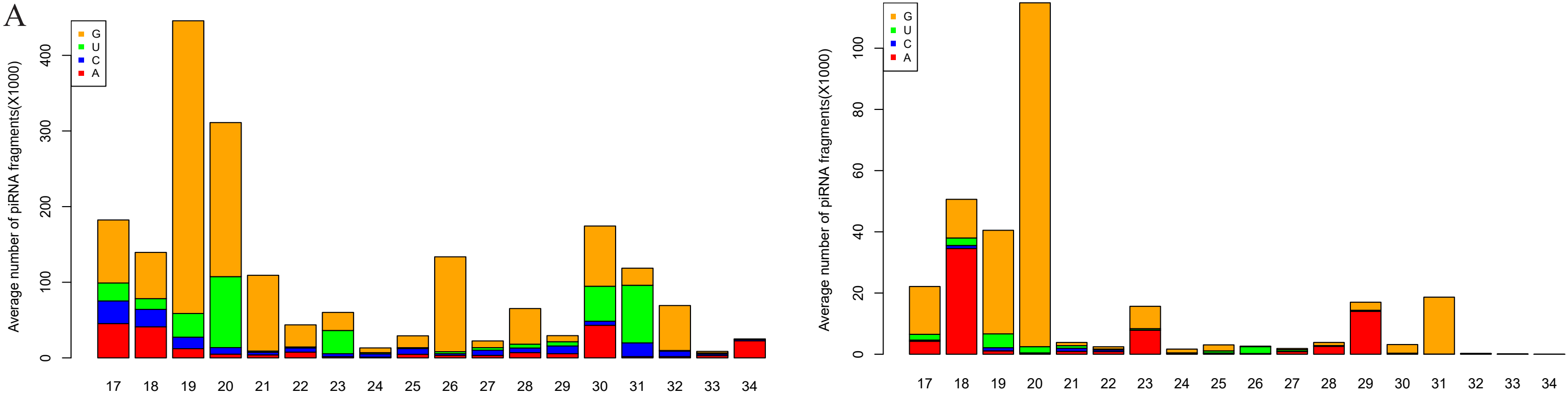
G
U
C
A
Average number of piRNA fragments(X1000)
0
100
200
300
400
17 18 19 20 21 22 23 24 25 26 27 28 29 30 31 32 33 34
G
U
C
A
Average number of piRNA fragments(X1000)
0
20
40
60
80
100
17 18 19 20 21 22 23 24 25 26 27 28 29 30 31 32 33 34

B

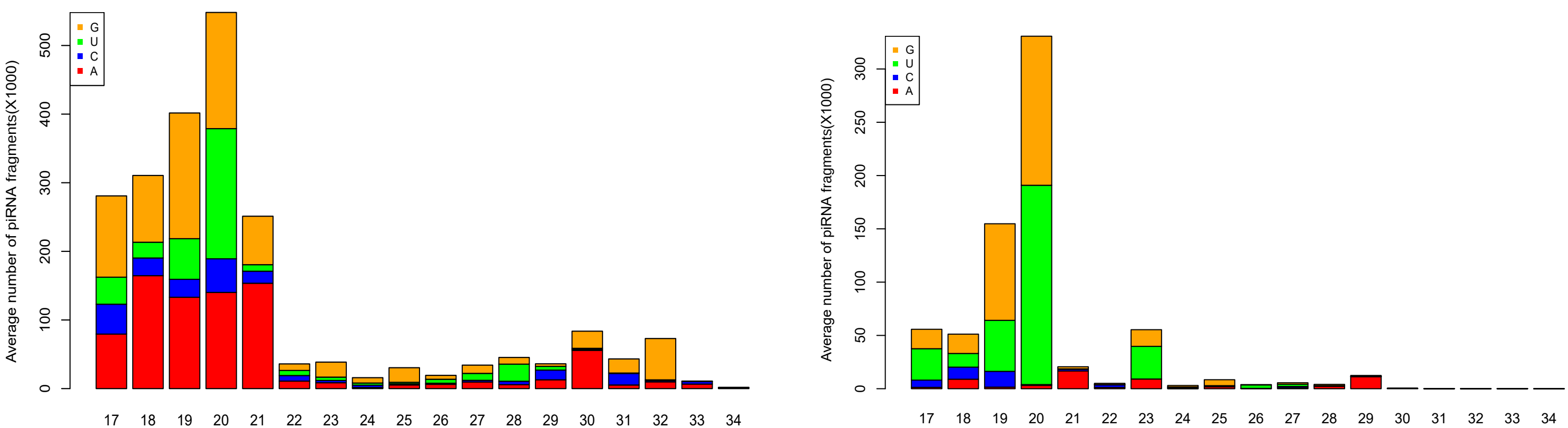
G
U
C
A
Average number of piRNA fragments(X1000)
0
100
200
300
400
500
17 18 19 20 21 22 23 24 25 26 27 28 29 30 31 32 33 34
G
U
C
A
Average number of piRNA fragments(X1000)
0
50
100
150
200
250
300
17 18 19 20 21 22 23 24 25 26 27 28 29 30 31 32 33 34

FigS6

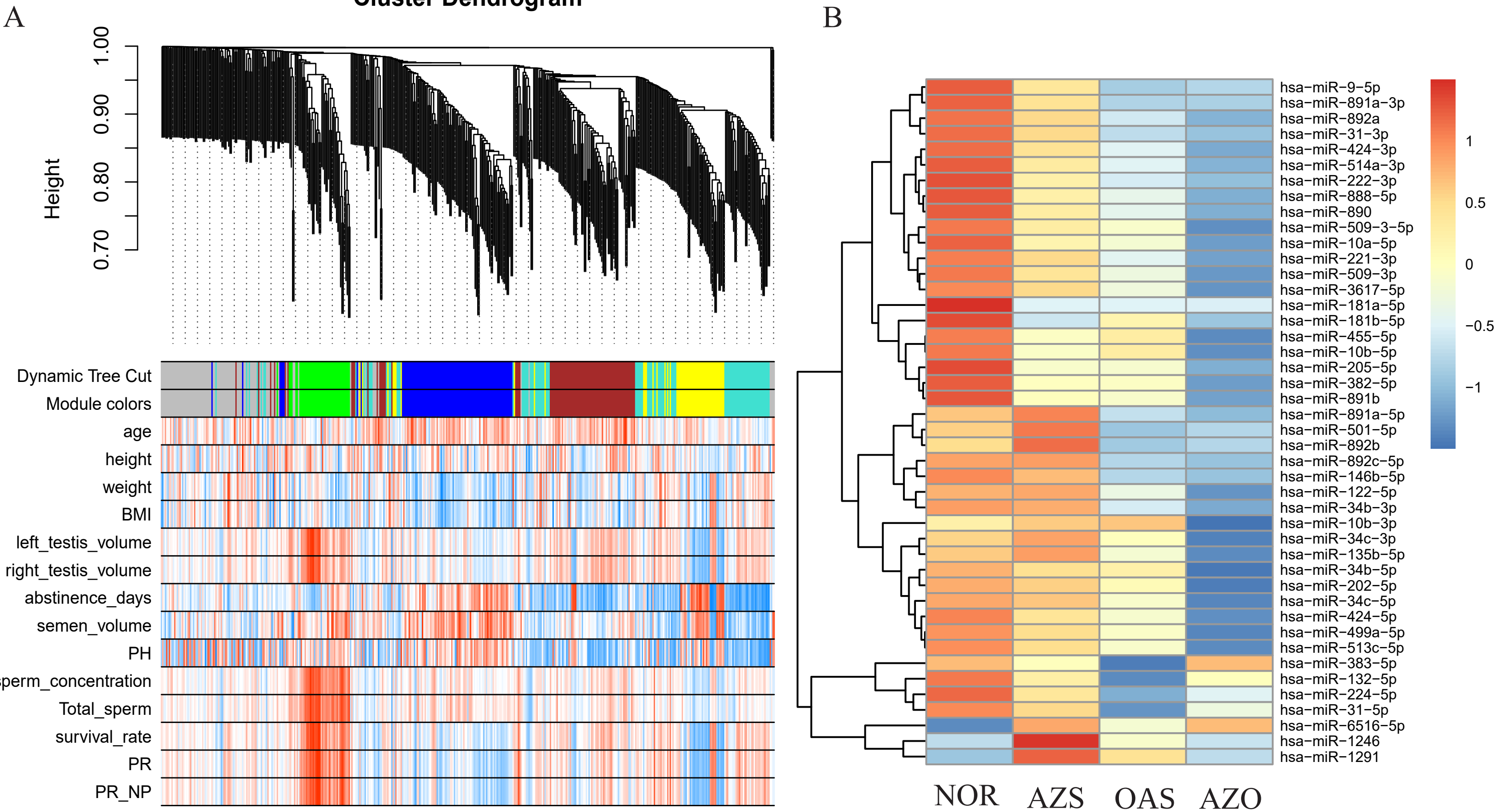
A
Cluster Dendrogram
Height
1.00
0.90
0.80
0.70
Dynamic Tree Cut
Module colors
age
height
weight
BMI
left_testis_volume
right_testis_volume
abstinence_days
semen_volume
PH
sperm_concentration
Total_sperm
survival_rate
PR
PR_NP
B
hsa−miR−9−5p
hsa−miR−891a−3p
hsa−miR−892a
hsa−miR−31−3p
hsa−miR−424−3p
hsa−miR−514a−3p
hsa−miR−222−3p
hsa−miR−888−5p
hsa−miR−890
hsa−miR−509−3−5p
hsa−miR−10a−5p
hsa−miR−221−3p
hsa−miR−509−3p
hsa−miR−3617−5p
hsa−miR−181a−5p
hsa−miR−181b−5p
hsa−miR−455−5p
hsa−miR−10b−5p
hsa−miR−205−5p
hsa−miR−382−5p
hsa−miR−891b
hsa−miR−891a−5p
hsa−miR−501−5p
hsa−miR−892b
hsa−miR−892c−5p
hsa−miR−146b−5p
hsa−miR−122−5p
hsa−miR−34b−3p
hsa−miR−10b−3p
hsa−miR−34c−3p
hsa−miR−135b−5p
hsa−miR−34b−5p
hsa−miR−202−5p
hsa−miR−34c−5p
hsa−miR−424−5p
hsa−miR−499a−5p
hsa−miR−513c−5p
hsa−miR−383−5p
hsa−miR−132−5p
hsa−miR−224−5p
hsa−miR−31−5p
hsa−miR−6516−5p
hsa−miR−1246
hsa−miR−1291
1
0.5
0
−0.5
−1
NOR
AZS
OAS
AZO

FigS7

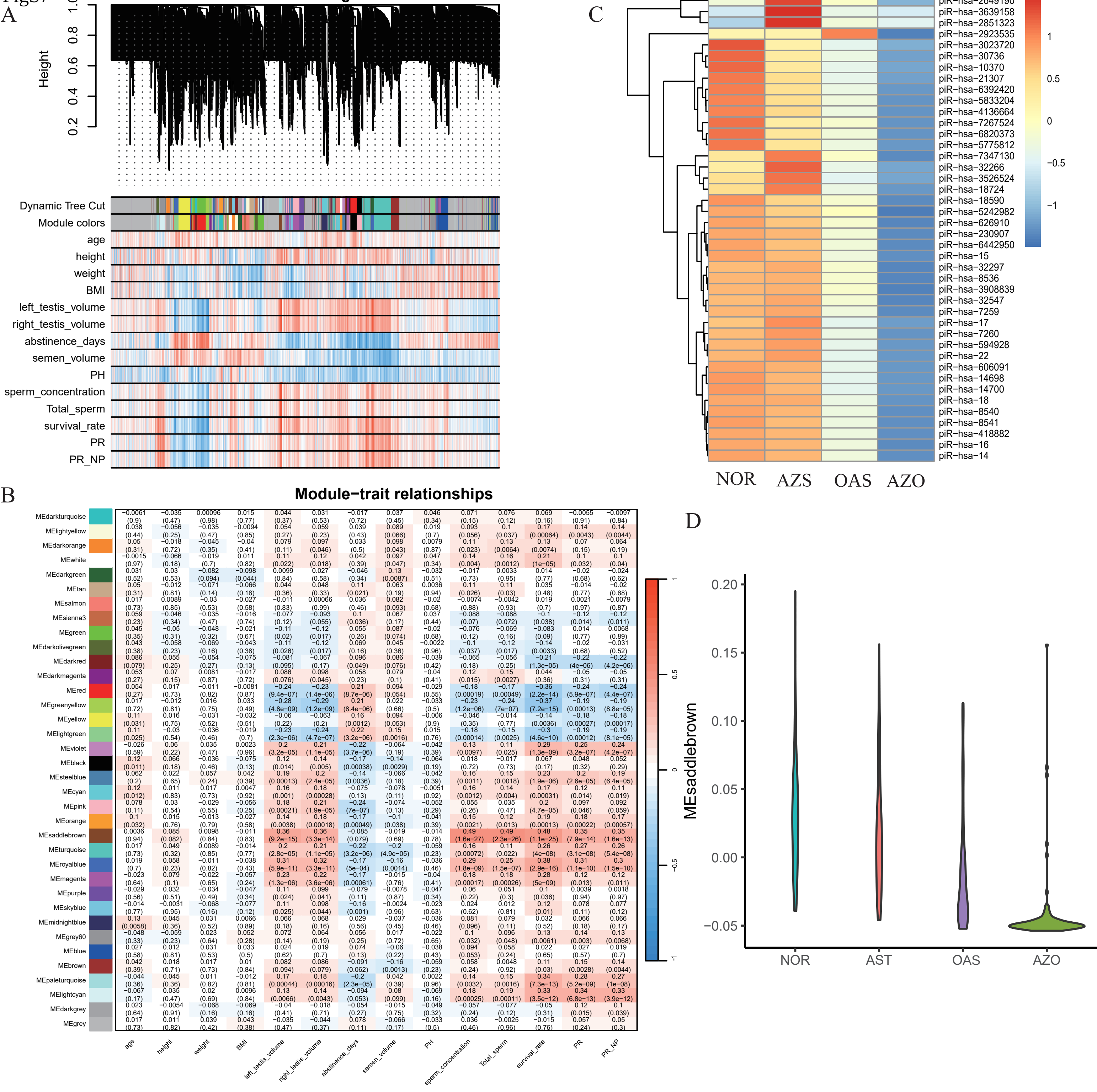

A
Cluster Dendrogram
Height
1.0
0.8
0.6
0.4
0.2
Dynamic Tree Cut
Module colors
age
height
weight
BMI
left_testis_volume
right_testis_volume
abstinence_days
semen_volume
PH
sperm_concentration
Total_sperm
survival_rate
PR
PR_NP
B
Module−trait relationships
MEdarkturquoise
MElightyellow
MEdarkorange
MEwhite
MEdarkgreen
MEtan
MEsalmon
MEsienna3
MEgreen
MEdarkolivegreen
MEdarkred
MEdarkmagenta
MEred
MEgreenyellow
MEyellow
MElightgreen
MEviolet
MEblack
MEsteelblue
MEcyan
MEpink
MEorange
MEsaddlebrown
MEturquoise
MEroyalblue
MEmagenta
MEpurple
MEskyblue
MEmidnightblue
MEgrey60
MEblue
MEbrown
MEpaleturquoise
MElightcyan
MEdarkgrey
MEgrey
age
height
weight
BMI
left_testis_volume
right_testis_volume
abstinence_days
semen_volume
PH
sperm_concentration
Total_sperm
survival_rate
PR
PR_NP
1
0.5
0
−0.5
−1
C
piR-hsa-2649190
piR-hsa-3639158
piR-hsa-2851323
piR-hsa-2923535
piR-hsa-3023720
piR-hsa-30736
piR-hsa-10370
piR-hsa-21307
piR-hsa-6392420
piR-hsa-5833204
piR-hsa-4136664
piR-hsa-7267524
piR-hsa-6820373
piR-hsa-5775812
piR-hsa-7347130
piR-hsa-32266
piR-hsa-3526524
piR-hsa-18724
piR-hsa-18590
piR-hsa-5242982
piR-hsa-626910
piR-hsa-230907
piR-hsa-6442950
piR-hsa-15
piR-hsa-32297
piR-hsa-8536
piR-hsa-3908839
piR-hsa-32547
piR-hsa-7259
piR-hsa-17
piR-hsa-7260
piR-hsa-594928
piR-hsa-22
piR-hsa-606091
piR-hsa-14698
piR-hsa-14700
piR-hsa-18
piR-hsa-8540
piR-hsa-8541
piR-hsa-418882
piR-hsa-16
piR-hsa-14
1
0.5
0
−0.5
−1
NOR
AZS
OAS
AZO
D
MEsaddlebrown
0.20
0.15
0.10
0.05
0.00
−0.05
NOR
AST
OAS
AZO

FigS8

A

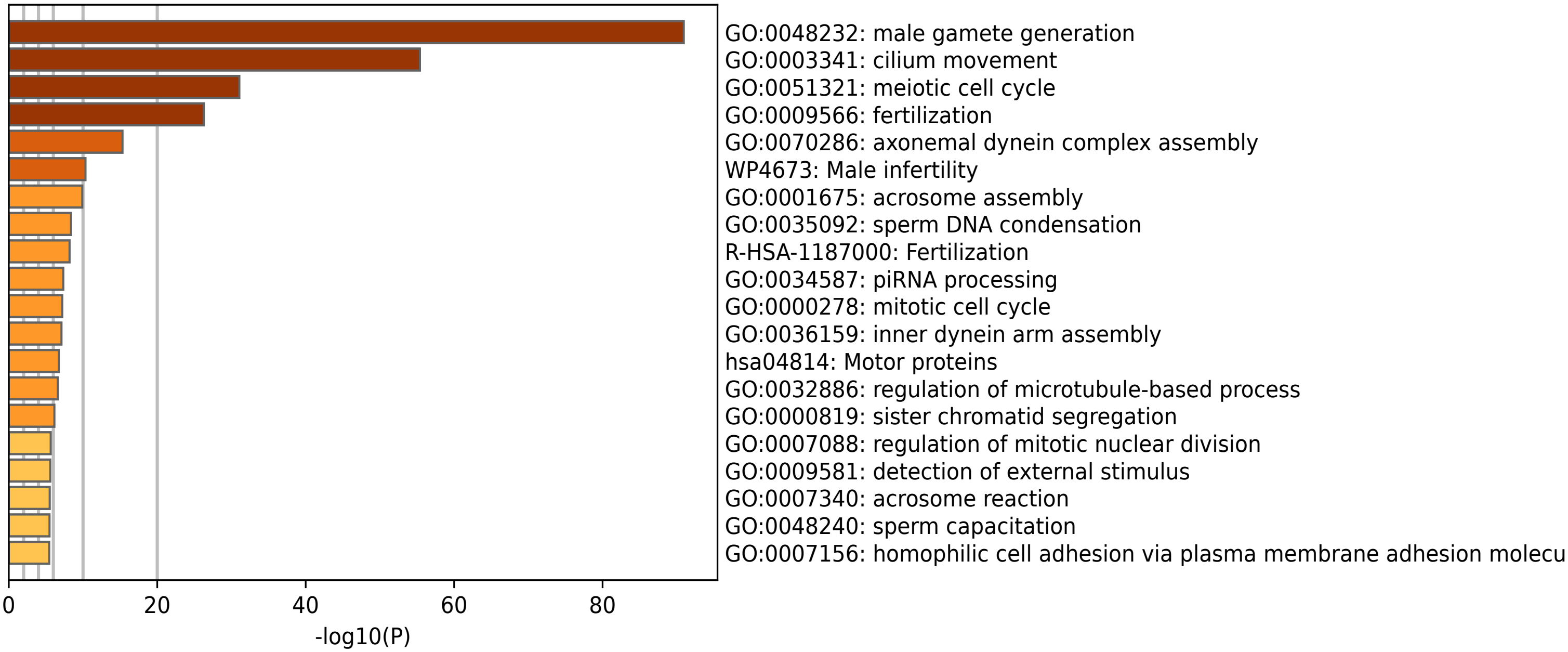
GO:0048232: male gamete generation
GO:0003341: cilium movement
GO:0051321: meiotic cell cycle
GO:0009566: fertilization
GO:0070286: axonemal dynein complex assembly
WP4673: Male infertility
GO:0001675: acrosome assembly
GO:0035092: sperm DNA condensation
R-HSA-1187000: Fertilization
GO:0034587: piRNA processing
GO:0000278: mitotic cell cycle
GO:0036159: inner dynein arm assembly
hsa04814: Motor proteins
GO:0032886: regulation of microtubule-based process
GO:0000819: sister chromatid segregation
GO:0007088: regulation of mitotic nuclear division
GO:0009581: detection of external stimulus
GO:0007340: acrosome reaction
GO:0048240: sperm capacitation
GO:0007156: homophilic cell adhesion via plasma membrane adhesion molecul
0
20
40
60
80
-log10(P)

B

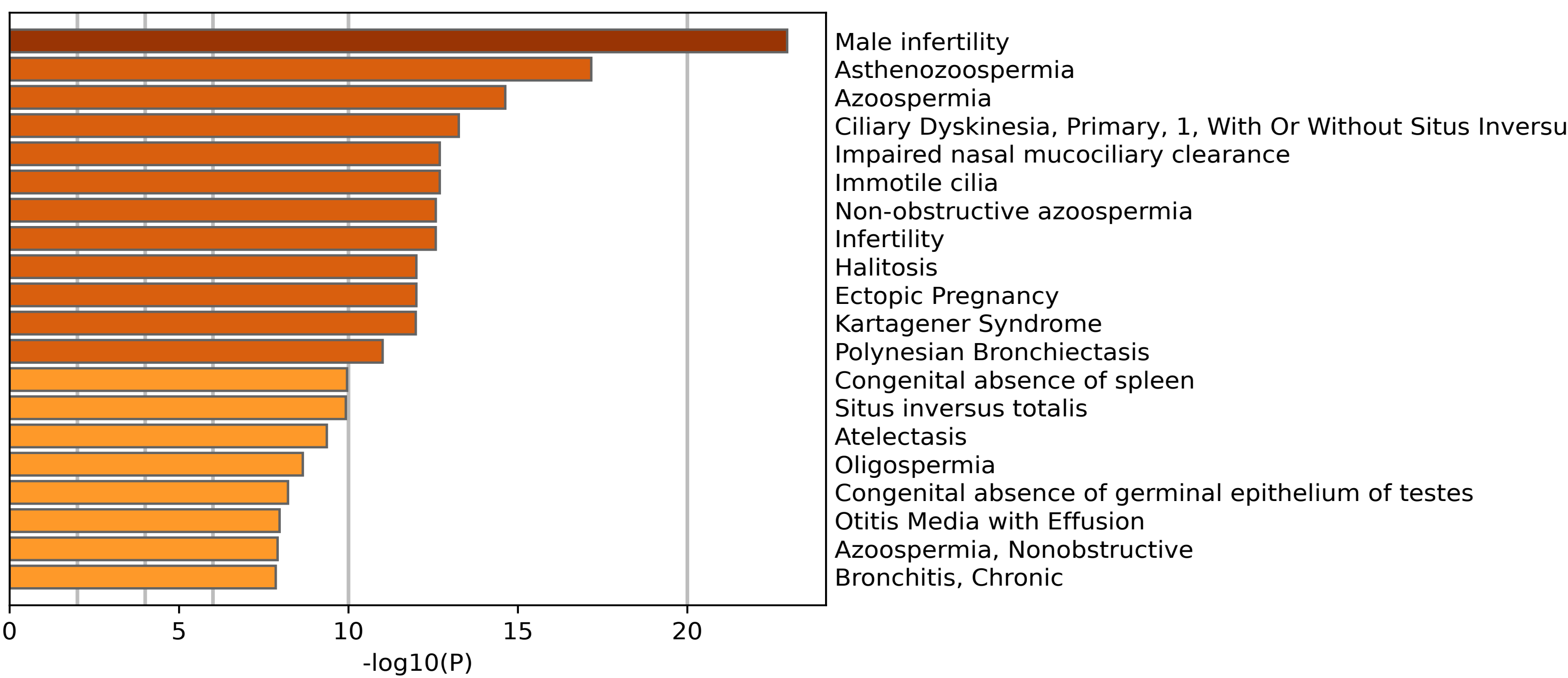
Male infertility
Asthenozoospermia
Azoospermia
Ciliary Dyskinesia, Primary, 1, With Or Without Situs Inversu
Impaired nasal mucociliary clearance
Immotile cilia
Non-obstructive azoospermia
Infertility
Halitosis
Ectopic Pregnancy
Kartagener Syndrome
Polynesian Bronchiectasis
Congenital absence of spleen
Situs inversus totalis
Atelectasis
Oligospermia
Congenital absence of germinal epithelium of testes
Otitis Media with Effusion
Azoospermia, Nonobstructive
Bronchitis, Chronic
0
5
10
15
20
-log10(P)

FigS9

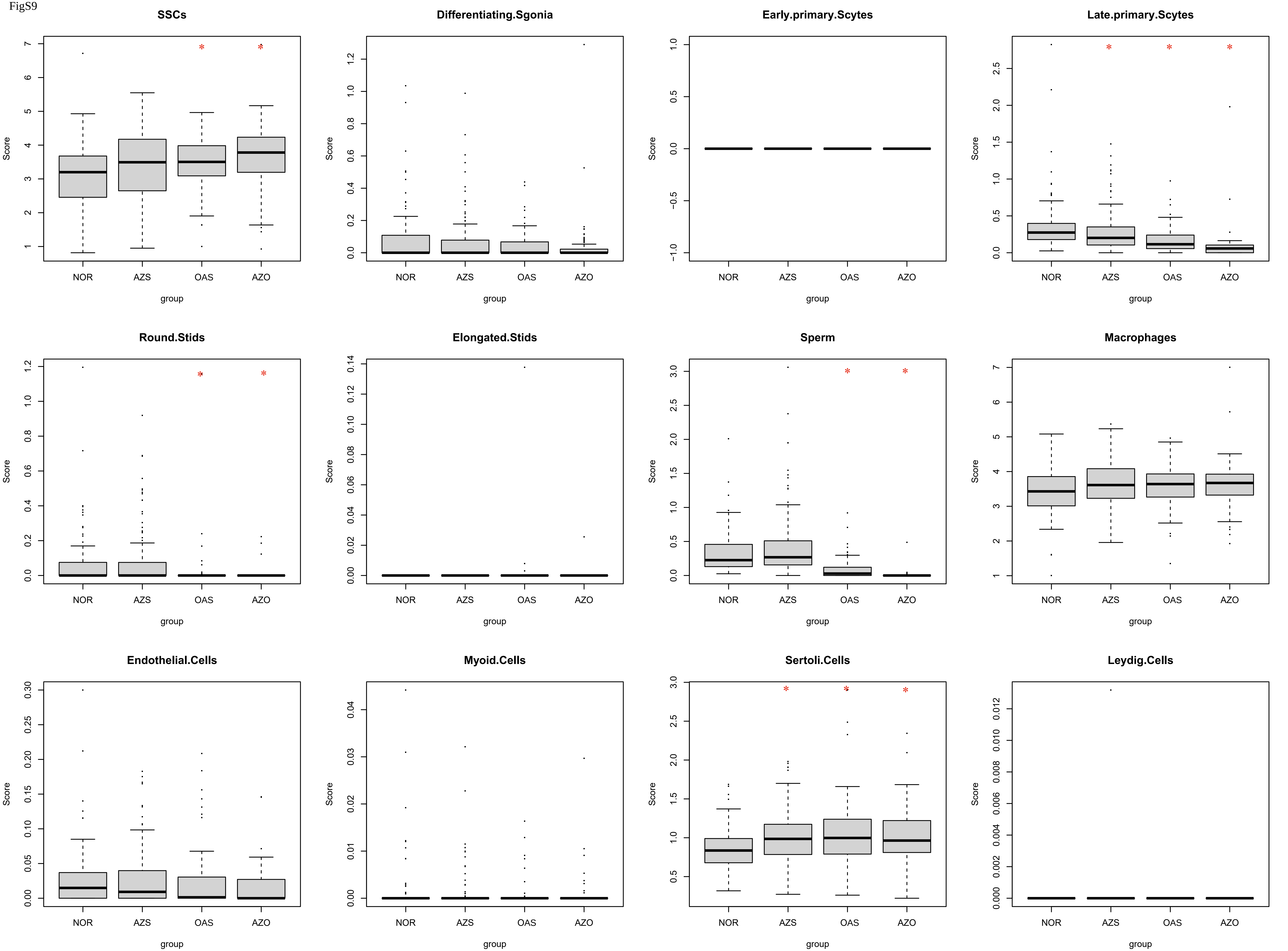
SSCs
Differentiating.Sgonia
Early.primary.Scytes
Late.primary.Scytes
Round.Stids
Elongated.Stids
Sperm
Macrophages
Endothelial.Cells
Myoid.Cells
Sertoli.Cells
Leydig.Cells
Score
group
NOR
AZS
OAS
AZO

FigS10

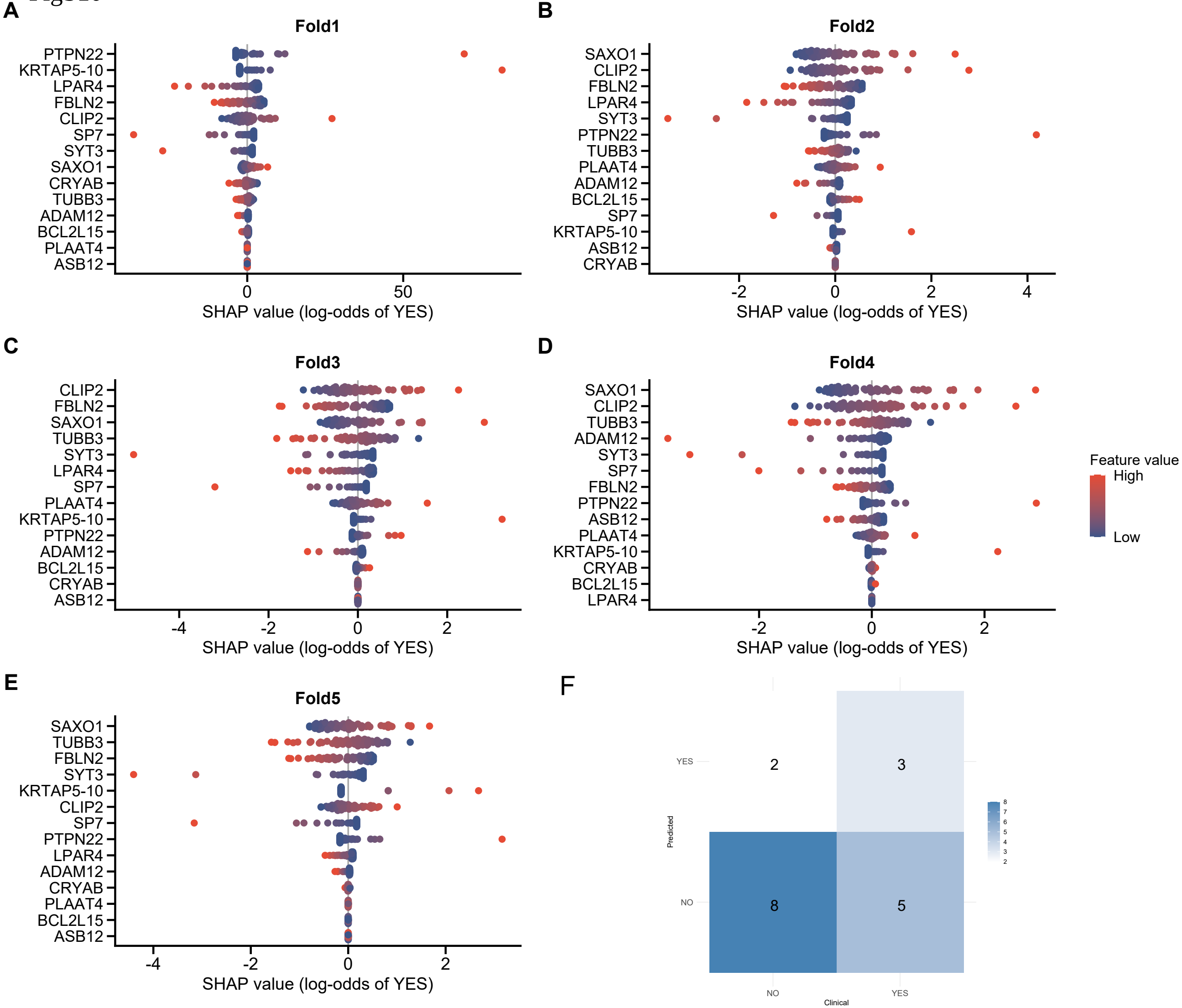

A
Fold1
PTPN22
KRTAP5-10
LPAR4
FBLN2
CLIP2
SP7
SYT3
SAXO1
CRYAB
TUBB3
ADAM12
BCL2L15
PLAAT4
ASB12
0
50
SHAP value (log-odds of YES)
B
Fold2
SAXO1
CLIP2
FBLN2
LPAR4
SYT3
PTPN22
TUBB3
PLAAT4
ADAM12
BCL2L15
SP7
KRTAP5-10
ASB12
CRYAB
-2
0
2
4
SHAP value (log-odds of YES)
C
Fold3
CLIP2
FBLN2
SAXO1
TUBB3
SYT3
LPAR4
SP7
PLAAT4
KRTAP5-10
PTPN22
ADAM12
BCL2L15
CRYAB
ASB12
-4
-2
0
2
SHAP value (log-odds of YES)
D
Fold4
SAXO1
CLIP2
TUBB3
ADAM12
SYT3
SP7
FBLN2
PTPN22
ASB12
PLAAT4
KRTAP5-10
CRYAB
BCL2L15
LPAR4
-2
0
2
SHAP value (log-odds of YES)
Feature value
High
Low
E
Fold5
SAXO1
TUBB3
FBLN2
SYT3
KRTAP5-10
CLIP2
SP7
PTPN22
LPAR4
ADAM12
CRYAB
PLAAT4
BCL2L15
ASB12
-4
-2
0
2
SHAP value (log-odds of YES)
F
YES
NO
Predicted
2
3
8
5
NO
YES
Clinical
8
7
6
5
4
3
2
