# Supplementary material for: Comprehensive profiling of cell‐free RNA in seminal plasma reveals functional links to spermatogenic impairment
Source: Clin Transl Med. 2026 Sep 1;16(9):e70788. doi: 10.1002/ctm2.70788 (PMC13535125; doi:10.1002/ctm2.70788)
Supplement: Supplementary file 2 [file CTM2-16-e70788-s003.docx]

### **Materials and methods**

## Ethic declaration

This study was approved and guided by the Medical Ethics Committee of the First Hospital of Jilin University (20K102-001). All procedures involving human participants were followed the ethical standards of the institutional committee and in accordance with the 1964 Helsinki declaration and its later amendments or comparable ethical standards.

## Recruitment of participants

Between April and November 2021, all participants were recruited from the Reproductive Medicine Center of the First Hospital of Jilin University following written informed consent. Individuals with normal findings on pre-pregnancy examination and routine semen analysis were recruited as healthy controls. Eligible infertile men were aged 22-45 years and comprised either couples meeting the clinical definition of infertility (failure to conceive after ≥1 year of unprotected intercourse). Semen samples were obtained by masturbation, and sperm concentration and motility were analyzed using a computer‑assisted sperm analysis (CASA) system (BEION S3, Beion Medical Technology Co., Ltd., Shanghai, China). All semen assessments and were performed in accordance with the fifth editions of the World Health Organization (WHO) laboratory manual guidelines^1^. Finally, individuals with lower sperm motility (progressive motility < 32% or total motility < 40%) and normal sperm count (≥39 × 10^6^ per ejaculate) were classified as AZS group. Men with lower sperm count (<39 × 10^6^ per ejaculate) were defined as OAS group, since they all have mixed pathologies of lower motility after recruitment, and men with no sperm were in AZO group. The parameter of sperm morphology was not considered here.

Among these recruited patients, some of them chose assisted reproductive technology (ART) for treatment. Only couples with no significant female factor were included, and ART was performed exclusively for male factor infertility. Successful ART outcome was defined as clinical pregnancy confirmed by transvaginal ultrasound showing intrauterine gestational sac and fetal heartbeat at 4 weeks after embryo transfer. Failed ART outcome was defined as negative serum hCG (<5 mIU/mL) at 14 days post-transfer or absence of pregnancy on ultrasound. A total of 77 participants with complete ART outcome data (first or second IVF/ICSI cycles) were included (34 success, 43 failure). Another follow-up information from 18 participants were collected as independent validation cohort.

## Seminal plasma processing

Following initial semen analysis, each sample was allowed to liquefy at 37 °C for 30 min. Seminal plasma was then separated by centrifugation at 3000 revolutions per minute(rpm) for 8 minute at 4 °C. To remove residual sperm and cellular debris, the supernatant was subjected to a second centrifugation step at 12,000 rpm for 10 min at 4 °C. The resulting cell‑free seminal plasma was transferred to cryopreservation tubes and promptly mixed with TRIzol LS reagent (Thermo Fisher Scientific) at a 1:3 (300 *µL* plasma: 900 *µL* reagent) ratio by vigorous vortexing. Aliquots were then stored at –80 °C until RNA extraction.

## Seminal RNA processing

RNA was extracted from 200 *µL* of thawed total seminal plasma using a Phenol-Chloroform RNA Extraction method^2^ according to the manufacturer’s protocol. Sequencing libraries were prepared with the PALM‑Seq method as previously described^3^. Libraries were then subjected to single‑end sequencing strategy with length of 100 base pair (bp) on a DNBSEQ‑T1 platform (MGI), generating an average of 130 million reads per sample.

## Data processing

Raw sequencing data were processed using the established PALM‑Seq pipeline^3^. After removing adapters and filtering short reads (< 17 nucleotide), sequencing reads were processed through sequential alignment steps to different RNA categories. Next, small‑RNA (miRNA, tRNA and piRNA) counts were normalized to reads per million mapped reads (RPM). For mRNAs and lncRNAs, expression levels were quantified as transcripts per million (TPM). After pre-processing and quality control, samples with extremely low number of clean reads (<10 million) were excluded for further analysis (FigureS2).

## Advanced analysis

To identify co‑expression modules of all RNA classes, we performed the weighted gene co-expression network analysis (WGCNA) using the WGCNA package in R. Differential abundance analysis was performed with DESeq2. RNAs exhibiting |log_2_(fold change)| ≥ 1 and an adjusted p‑value < 0.05 (Bonferroni–Holm correction) were considered as RNA with different abundant and RNAs with no abundance in ≥ 70% of samples were filtered out. The Gene Set Enrichment Analysis (GSEA) was performed to identify biological pathways and processes systematically enriched within the gene sets. Processes with a nominal p-value < 0.05 were considered statistically significant. Cellular deconvolution of testicular cell‑type contributions was performed with CIBERSORTx^4^, using a public dataset of human testis single-cell RNA-seq (scRNA‑seq)^5^ as input reference expression matrix. Pre‑pachytene and pachytene piRNAs were classified according to genomic index if it overlapped with annotated loci from Ozata *et al*^6^. The competing endogenous RNA (ceRNA) networks were identified with the R package GDCRNATools, based on both reported targeting regulation and associated expression pattern between RNA members. Expression of ceRNA network components in normal human testicular cells was provided by HumanTestisdb (https://shalab.njmu.edu.cn/humantestisdb/) and their expression in infertile individual’s germ cells were generated by a published dataset from Mahyari *et al*^7^ (https://conradlab.shinyapps.io/HISTA/).

## Regression modelling of semen parameters

### Cohort definitions

Three independent regression models were developed to predict distinct semen parameters-total sperm number, progressive motility (PR), and total motility (TM). The full cohort of 419 samples was utilized in the model of sperm number, encompassing the entire spectrum of sperm concentrations. Given that sperm motility is biologically undefined in azoospermic samples and is heavily confounded by severe oligozoospermia, the PR and TM models were restricted to 229 samples with normal sperm number ( ≥ 39 × 10⁶ per ejaculate), which comprised the normozoospermic (NOR) and AZS group.

### Cross-validation design

For each target, samples were split into five folds with caret::createFolds (k = 5), so that every sample served exactly once as an out-of-fold test set while the remaining four folds were used for feature selection and model training. The same five-fold partition was shared by all three targets: it was defined once on the full-cohort sample set of the number model, and the PR and TM models inherited this partition restricted to their respective subsets of samples with a normal sperm number, so that every sample was always assigned to the same fold across the three models. All feature selection, hyperparameter tuning, and preprocessing estimation were performed exclusively within each training partition to prevent information leakage.

### Bootstrap consensus feature selection

Within each training partition, feature stability was assessed with 50 stratified bootstrap resamples. In each resample, genes were firstly filtered by Pearson correlation with the target (Benjamini–Hochberg FDR < 0.01 and |r| > 0.3) and were then subjected to two complementary selectors: LASSO regression (glmnet; genes with non-zero coefficients retained) and a random forest (RF, ranger; top-ranking genes by variable importance retained). Selection counts were tallied across the 50 bootstrap resamples, and genes were ranked by the combined count of LASSO and RF. The top n genes were taken as candidate feature sets, with n ∈ {5, 10, 15, 20, 25, 30}.

### Model training and hyperparameter tuning

Six regression algorithms were benchmarked for every feature-set size: elastic-net linear regression (glmnet), linear/polynomial/radial-basis-function support vector machines (kernlab), random forest (ranger), and gradient-boosted trees (XGBoost). Predictors were z-score standardised within each training fold. Model hyperparameters were tuned by internal cross-validation within each training fold, and the final values were selected with the one-standard-error rule, which favours simpler models.

### Model selection and evaluation

For each algorithm × feature-set-size combination, performance was summarised as the mean Pearson correlation coefficient (r) across the five out-of-fold test sets, and the combination with the highest mean r was retained as the optimised model. Random forest gave the best performance for all three targets, with 30 features for number and 20 features for PR and TM. Final performance was reported on the pooled out-of-fold predictions as Pearson r, coefficient of determination (R²), and RMSE; negative predictions were truncated at zero. For clinical interpretation, observed and predicted values were converted into diagnostic categories using WHO thresholds: sperm number < 39 × 10⁶ as abnormal; PR ≥ 32% and TM ≥ 40% as normal motility. Classification agreement was summarised by a Sankey diagram linking number-based predictions, reference clinical groups, and motility-based predictions.

## Classification modelling of ART outcome

### Feature set

No data-driven feature selection was performed for the classification task. Instead, 15 mRNAs were nominated a priori from biological knowledge, of which 14 passed the expression-detection filter (NECTIN2 was excluded owing to low detection) and were used as model features: *TUBB3, SAXO1, FBLN2, CLIP2, BCL2L15, ASB12, CRYAB, PLAAT4, KRTAP5-10, SYT3, PTPN22, SP7, ADAM12*, and *LPAR4*.

### Model training and tuning

The 77 samples were split into five folds with caret::createFolds (k = 5, stratified by outcome). The same six algorithm families as above were benchmarked in classification mode: elastic-net logistic regression (glmnet), three SVM variants (kernlab), random forest (ranger), and XGBoost. Since the two classes were imbalanced (34 YES vs 43 NO), the majority class was randomly downsampled to a 1:1 ratio within each training fold only (themis::step_downsample), followed by z-score standardisation of predictors. Model hyperparameters were tuned by internal cross-validation within each training fold and selected with the one-standard-error rule.

### Performance evaluation and model interpretation

Classification performance was evaluated on the out-of-fold test sets using the area under the receiver operating characteristic curve (ROC AUC) and the area under the precision–recall curve (PR AUC, with the class prevalence as baseline). Elastic-net logistic regression achieved the best performance (test ROC AUC 0.833 ± 0.137; PR AUC 0.826 ± 0.126, mean ± SD across folds). ROC curves were computed with the *pROC* package; AUC confidence intervals (CIs) were estimated by the DeLong method, and the 95% CI band of the pooled test ROC curve was estimated from 2,000 stratified bootstrap replicates. The probability threshold for binary classification was determined by maximising Youden's index on the pooled test ROC curve. The follow-up 18 samples were used for external evaluation of the trained workflows; per-sample probabilities were averaged across the five fold-specific models and converted into class calls with the Youden threshold.

To interpret the five fold-specific logistic-regression models, Shapley additive explanations (SHAP)^8^ were computed exactly with the *kernelshap* package, enumerating all 2¹⁴ feature coalitions, using each fold's preprocessed training data as the background dataset and predicting on the log-odds scale. Feature importance was summarised as the mean absolute SHAP value per mRNA and visualised with *shapviz* beeswarm plots.

## Software and statistical analysis

All analyses were performed in R v4.5.0 (aarch64-apple-darwin). Model training and evaluation used the *tidymodels* framework (recipes, parsnip, tune, yardstick, rsample, workflows) with engines *glmnet*, *kernlab*, *ranger*, and *xgboost*; resampling utilities from *caret*; class-imbalance handling from *themis*; and variable-importance extraction from *vip*. Variance partitioning used *variancePartition* v1.41.4. ROC analysis used *pROC*. Model interpretation used *kernelshap* (exact algorithm) and *shapviz*. Multiple-testing correction used the Benjamini–Hochberg procedure. Two-sided P < 0.05 was considered statistically significant unless otherwise stated.

# Reference

1.	World Health Organization. WHO Laboratory Manual for the Examination of Human Semen and Sperm–Cervical Mucus Interaction 5th edn. Cambridge Univ. Press.

2.	Toni LS, Garcia AM, Jeffrey DA, et al. Optimization of phenol-chloroform RNA extraction. MethodsX. 2018;5:599-608. doi:10.1016/j.mex.2018.05.011

3.	Liu Z, Wang T, Yang X, et al. Polyadenylation ligation‐mediated sequencing (PALM‐Seq) characterizes cell‐free coding and non‐coding RNAs in human biofluids. Clinical and Translational Medicine. 2022;12(7)doi:10.1002/ctm2.987

4.	Newman AM, Steen CB, Liu CL, et al. Determining cell type abundance and expression from bulk tissues with digital cytometry. Nat Biotechnol. Jul 2019;37(7):773-782. doi:10.1038/s41587-019-0114-2

5.	Guo J, Grow EJ, Mlcochova H, et al. The adult human testis transcriptional cell atlas. Cell Res. Dec 2018;28(12):1141-1157. doi:10.1038/s41422-018-0099-2

6.	Özata DM, Yu T, Mou H, et al. Evolutionarily conserved pachytene piRNA loci are highly divergent among modern humans. Nature ecology & evolution. Jan 2020;4(1):156-168. doi:10.1038/s41559-019-1065-1

7.	Mahyari E, Guo J, Lima AC, et al. Comparative single-cell analysis of biopsies clarifies pathogenic mechanisms in Klinefelter syndrome. American journal of human genetics. Oct 7 2021;108(10):1924-1945. doi:10.1016/j.ajhg.2021.09.001

8.	Lundberg SM, Erion G, Chen H, et al. From local explanations to global understanding with explainable AI for trees. Nature Machine Intelligence. 2020/01/01 2020;2(1):56-67. doi:10.1038/s42256-019-0138-9
