# Supplementary material for: Comprehensive profiling of cell‐free RNA in seminal plasma reveals functional links to spermatogenic impairment
Source: Clin Transl Med. 2026 Sep 1;16(9):e70788. doi: 10.1002/ctm2.70788 (PMC13535125; doi:10.1002/ctm2.70788)
Supplement: Supplementary file 3 [file CTM2-16-e70788-s007.docx]

Table 1. Clinical characteristics of recruited individuals.

| **characteristic** | **NOR**  **101** | **AZS**  **128** | **OAS**  **93** | **azo**  **97** |
| --- | --- | --- | --- | --- |
| **Age, years**  Mean  Range | 31.05  [21,42] | 32.9  [23,55] | 32.16  [20,48] | 31.49  [21,47] |
| **BMI**  MEAN  RANGE | 26.02  [15.85,37.04] | 25.83  [17.31,37.55] | 26.68  [17.92,40.74] | 24.92  [15.73,35.49] |
| **Total sperm number(10^6 per ejaculate)**  MEAN  RANGE | 335.8  [41.6,887.2] | 208.8  [32.2,736.4]  *** | **8.604**  [0.5,30.2]  *** | **0** |
| **Progressive motility(PR,%)**  MEAN  RANGE | 56.22  [21.1,89.6] | **5.99**  [0,51.2]  *** | 12.7  [0,52.8]  *** | 0 |
| **Total motility(PR+NP,%)**  mean  range | 59.81  [21.7,92.4] | **6.567**  [0,53,4]  *** | 15.65  [0,55.6]  *** | 0 |
| **46,XXY** | - | - | - | 4/97 |

*** p<0.0001
